# Epidemiological Characterization of African Swine Fever Dynamics in Ukraine, 2012–2023

**DOI:** 10.3390/vaccines11071145

**Published:** 2023-06-25

**Authors:** Maksym Bezymennyi, Oleksandr Tarasov, Ganna V. Kyivska, Nataliia A. Mezhenska, Svitlana Mandyhra, Ganna Kovalenko, Mykola Sushko, Nataliia Hudz, Serhii V. Skorokhod, Roman Datsenko, Larysa Muzykina, Elaina Milton, Maryna A. Sapachova, Serhii Nychyk, Ihor Halka, Maciej Frant, Falk Huettmann, Devin M. Drown, Anton Gerilovych, Andrii A. Mezhenskyi, Eric Bortz, Christian E. Lange

**Affiliations:** 1 Institute of Veterinary Medicine (IVM), National Academy of Agrarian Sciences of Ukraine, 03151 Kyiv, Ukraine; 2 State Scientific Research Institute of Laboratory Diagnostics and Veterinary and Sanitary Expertise (SSRILDVSE), 03151 Kyiv, Ukraine; 3 Department of Biological Sciences, University of Alaska Anchorage, Anchorage, AK 99508, USA; 4 Department of Swine Diseases, National Veterinary Research Institute (NVRI), 24-100 Pulawy, Poland; 5 Institute of Arctic Biology, University of Alaska Fairbanks, Fairbanks, AK 99775, USA; 6 Department of Biology and Wildlife, University of Alaska Fairbanks, Fairbanks, AK 99775, USA; 7 Metabiota Inc., San Francisco, CA 94104, USA; 8 Labyrinth Global Health, Saint Petersburg, FL 33704, USA; 9 Department of Biology, Kwantlen Polytechnic University, Surrey, BC V3W 2MB, Canada

**Keywords:** African swine fever, African swine fever virus, ASF, ASFV, Ukraine, epidemiology, wild boar, backyard farms, outbreak, One Health

## Abstract

African swine fever (ASF) is a viral disease, endemic to Africa, that causes high mortality when introduced into domestic pig populations. Since the emergence of p72-genotype II African swine fever virus (ASFV) in Georgia in 2007, an ASF epidemic has been spreading across Europe and many countries in Asia. The epidemic first reached Ukraine in 2012. To better understand the dynamics of spread of ASF in Ukraine, we analyzed spatial and temporal outbreak data reported in Ukraine between 2012 and mid-2023. The highest numbers of outbreaks were reported in 2017 (N = 163) and 2018 (N = 145), with overall peak numbers of ASF outbreaks reported in August (domestic pigs) and January (wild boars). While cases were reported from most of Ukraine, we found a directional spread from the eastern and northern borders towards the western and southern regions of Ukraine. Many of the early outbreaks (before 2016) were adjacent to the border, which is again true for more recent outbreaks in wild boar, but not for recent outbreaks in domestic pigs. Outbreaks prior to 2016 also occurred predominantly in areas with a below average domestic pig density. This new analysis suggests that wild boars may have played an important role in the introduction and early spread of ASF in Ukraine. However, in later years, the dynamic suggests human activity as the predominant driver of spread and a separation of ASF epizootics between domestic pigs and in wild boars. The decline in outbreaks since 2019 suggests that the implemented mitigation strategies are effective, even though long-term control or eradication remain challenging and will require continued intensive surveillance of ASF outbreak patterns.

## 1. Introduction

African swine fever (ASF) is a highly contagious disease of domestic and wild *suids* (pigs), and it can be devastating for the farming economy. Originally restricted to Africa, there have since been sporadic outbreaks in other parts of the world. The first cases of ASF in China were reported in August 2018. In 2019, ASF killed about 25% of the world’s hogs. Clinically, ASF manifests as a hemorrhagic disease that is caused by the African swine fever virus (ASFV), a unique virus establishing the taxonomic categories of genus (*Asfivirus*), family (*Asfarviridae*) and order (*Asfuvirales*) as a sole member [1]. The large double-stranded DNA virus has a reservoir host in African soft ticks (*Onithodorus moubata*) and naturally infects wild African suids, such as bushpigs (*Potamochoerus porcus*), giant forest hogs (*Hylochoerus meinertzhageni*) and warthogs (*Phacochoerus aethiopicus*) [2,3,4,5,6,7,8,9]. While wild African suids commonly suffer only from mild effects or lack clinical symptoms, ASF outbreaks in wild boar (*Sus scrofa*) and especially in domestic pig (*Sus scrofa domestica*) populations are often devastating, with a very high case fatality rate [1]. Virulence appears to be strain-dependent, and high, moderate and low virulent strains have been described. Strains with low virulence were only described in regions where ASF had become endemic, such as the Iberian Peninsula in the 1950s and 1960s where initially highly virulent strains had circulated. Recent data from the Baltic states, especially from Estonia, indicate that there is a growing population of ASF survivors among wild boars, which might also indicate the presence of a strain with reduced virulence [10]. Since no vaccine or treatment is available to date, transmission control is the only effective line of intervention [1].

While endemic to Africa, ASF has on occasion been introduced to other regions, such as Europe in the 1950s and 1960s, as well as South America and the Caribbean in the 1970s. These outbreaks were eventually stopped by coordinated and rigorous interventions, and ASFV again was confined to Africa, with the only exception being the Italian island of Sardinia [11,12]. However, a virulent lineage of ASFV p72-genotype II was detected in Georgia in 2007, and this latest and still ongoing epizootic of ASF outside of Africa has since spread widely over Eurasia and caused enormous losses to the pork farming industry from eastern Europe, including the Russian Federation, Ukraine and Poland, to Southeast Asia, involving China, Mongolia, Vietnam, South Korea and the Philippines and other countries [13,14,15,16,17,18,19,20,21,22,23].

In Europe, ASF has progressed westward in a slow wave advancing through the wild boar population with spillover into domestic pig populations in all sorts of farming systems [1,19,24,25]. Infectious ASFV can survive for an extended period in the environment, such as in carcasses and on fomites, but also remains infectious in meat products made from infected animals and thus can inadvertently be carried long distances and across borders by human travel activities [1,26,27,28,29,30,31]. This makes ASFV very versatile in its modes of spread, and, depending on the country, the epidemiological situation and outbreak dynamics can differ significantly. Targeted control measures involving intense testing and reduction in wild boar populations, on the one side, and improved biosafety for farms, information campaigns and incentives on the domestic side of the equation have slowed down the spread in many places, but ASF continues to be on the move and threatens the pork industry around the globe [19,24,32].

After Ukraine had been considered as ASFV-free for 35 years, ASF reached Ukraine again in an isolated outbreak in 2012 [33]. Molecular sequencing of multiple isolates from Ukraine confirmed the current ASFV outbreak strain to belong to p72-genotype II, thus being most likely connected with the current ASF wave emanating from Georgia in 2007. Indeed, in our previous work, we found an ASFV genome from a domestic pig in Kyiv region, 2016, to be 99.9% identical to the virulent p72-genotype II strain isolated in Georgia in 2007 [18]. In the absence of a vaccine, understanding the epidemiology and interrupting the transmission routes wherever possible are the only feasible measures to control and fight ASF. This study was accordingly initiated to improve our understanding of the ASF situation and dynamics in Ukraine, where the first case was detected in 2012. Our goals were (a) to understand the probable source of introduction and how often introduction occurred; (b) to determine whether domestic pigs or wild boars were the likely source of ASF being imported into Ukraine; (c) to assess the risk based on the type of farming practice (holding form).

## 2. Materials and Methods

### 2.1. Data Sources 

Official data collected in Ukraine by the State Service of Ukraine for Food Safety and Consumer Protection (FSCP) between 2012 and 2023 were used for the epidemiological analysis (https://www.asf.vet.ua/, accessed on 8 May 2023). Data included the date of outbreak, geographical coordinates of the location, type of affected animal (wild, domestic or disposed carcasses or infected objects) and holding form (commercial farm, backyard farm) and total infected or suspected exposed animals for all reported ASF outbreaks in Ukraine. All outbreaks were confirmed by PCR testing for ASFV.

The incidence/risk of outbreaks by affected animal and holding form was analyzed in relation to total animals affected and total holdings or sounders affected. Based on data from the Agriculture in Ukraine Statistical Yearbook 2017 published by the State Statistic Service of Ukraine, we calculated with 2281 commercial farms, 2,254,000 backyard farms and 30,000 wild boars in Ukraine. Wild boars were assumed to be roaming in sounders of 5 adults on average.

### 2.2. Spatio-Temporal Epidemiology and Geographic Information System (GIS) Analyses

To characterize geographical areas of high and low outbreak activity, we performed optimized hot spot analysis from Spatial statistic toolset in ArcGIS (ESRI Inc., Redlands, CA, USA, version 10.4) for desktop. The tool generates a map of statistically significant hot and cold spots using the Getis-Ord Gi* statistic. [34]. For the analysis, we chose to aggregate outbreaks within a hexagonal grid.

Two kinds of temporal and spatial analysis were performed to characterize and visualize the ASF outbreak dynamics in Ukraine: a space–time permutation model from SatScan version 9.6 software (www.satscan.org) and one based on assumptions about ASF biology and behavior. The space–time permutation model uses coordinates and time of each event to test for statistically significant spatio-temporal clustering [34]. The maximum scanning window for the analysis was set to 60 days in time and 200 km in space. Time aggregation option was not applied. The maximum number of Monte Carlo replications was set to 999.

For the ASF biology and behavior model, outbreak clusters were identified as follows: outbreaks were considered connected to a previous outbreak if they occurred within 60 days (3 × maximum survival of acute form of ASF) of one another and were within 200 km (>10 × average diameter of male wild boar territory), which would be more than 20 times the speed of purely wild-boar-driven ASF spread [35,36]. These criteria would primarily connect outbreaks in which live animals transmit ASF directly, but they take into account the possibility that a significant proportion of outbreaks (2/3) in a particular infection chain may not be detected or reported. In cases where a new outbreak fell within the 60 days and 200 km of two or more previous outbreaks, it was counted as a continuation of the closer previous outbreak if either of two conditions were met: (A) the distance from the potential sources differed by more than 100 km or (B) the new outbreak was less than 50 km from one potential source but more than 100 km from the other(s). If neither condition was met, then the new outbreak was counted as a merger of two or more previous ones.

The velocity (speed in km/day) of ASF dissemination was evaluated individually for each cluster that included more than one reported outbreak based on the time between the initial report and the reported point furthest away from it.

In order to evaluate the dynamics behind the emergence and spread of ASF in Ukraine, distances to borders, major roads and urban centers were determined and analyzed for all reported outbreak locations. The closest direct distance from each reported outbreak to the next closest official land border (as of 2013) or the northern border of Crimea was determined using Google Maps and the average plotted for each year (April–March) starting in 2014. Google Maps tools were used to determine the distance between the outbreak sites (distance between two points) and major roads as well as to determine the driving distance (shortest route) to the next major city (>50,000 population based on 2014 estimates). Statistical significance of outbreak dynamics among groups was analyzed by one-way ANOVA and Student’s *t*-test using GraphPad 7.0.

Forest coverage, an indirect indicator of possible wild boar density (habitat) and thus for the potential of transmission through wild boars, within a radius of 25 km around outbreak sites was evaluated based on forest databases [37,38,39].

To evaluate the domestic pig population around each outbreak site, 25 km radius circles were drawn using the data of the Gilbert dataset as a basis [40].

To account for a potential influence of climatic factors, the territory of Ukraine was divided into two parts, the southern Black-Sea-adjacent oblasts (excluding the Crimean Peninsula), Kherson, Odesa and Mykolaiv on the one side, and the more northern other oblasts on the other, based on the Köppen climate classification (Figure S1). Wild boar and domestic pig cases were analyzed separately on a month by month basis.

## 3. Results

### 3.1. Seasonal Trends in ASF Outbreaks in Ukraine

**Figure 1:**
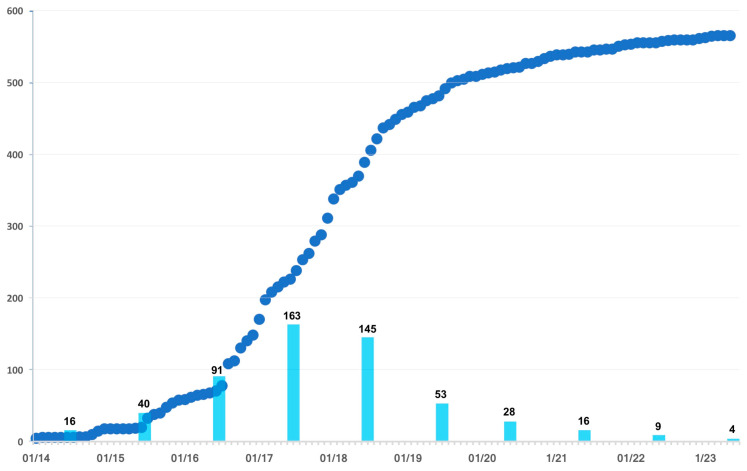
Number of reported ASF cases in Ukraine, depicted on a cumulative monthly basis (dark blue dots) and on a yearly basis (light blue bars) from January 2014 through May 2023.

Between the first detection of ASF in Ukraine in 2012 and the early May 2023, 566 outbreaks were reported, with over 300,000 domestic pigs in commercial and backyard farms being affected, and a small number of wild boars reported as positive for ASF from opportunistic sampling during hunting seasons (N = 125) (Figure S2). While ASF outbreak reports were most frequent in 2017 and 2018, with one-hundred-sixty-three and one-hundred-forty-five reports, respectively, they have decreased since, with fifty-three in 2019, eighty-two in 2020, sixteen in 2021, nine in 2022 and four in the first 4 months of 2023 (Figure 1).

**Figure 2:**
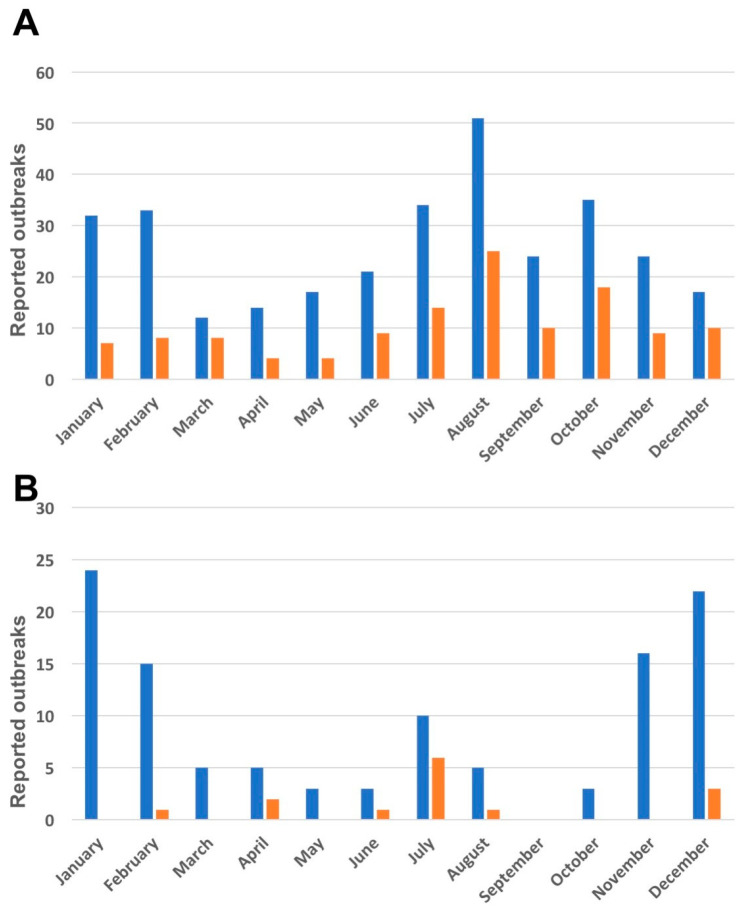
Reported outbreaks by month (cumulative number across all years) broken down by southern (Kherson, Odesa, Mykolaiv; orange bars) and northern (all other; blue bars) oblasts (excluding Crimea); panel (**A**) showing domestic pig and panel (**B**) wild boar outbreaks.

The months with the most reported outbreaks varied widely between wild and domestic swine (Figure 2). The month with the highest rate of outbreaks in domestic pigs was August (18.9%), and represents the peak of a June-to-October surge of domestic ASF outbreaks that was predominant in the northern oblasts (province-level administrative districts) of Ukraine but mirrored in southern Ukraine in Kherson, Odesa and Mykolaiv oblasts (Figure 2). The patterns (months with relatively high or low numbers of reported outbreaks) for domestic pigs varied between the northern and southern oblasts, with high numbers in the months July through October in both north and south, with additional peaks in January and February in northern oblasts. Meanwhile, ASF outbreaks in wild boars occurred predominantly in northern oblasts of Ukraine in the winter months of November through February, with a peak rate in December (21%). April was the month with the least reported outbreaks in domestic pigs (4.5%), May for domestic pigs and wild boars combined (4.2%) and September in the case of wild boars (0%).

### 3.2. Spatio-Temporal Analysis of ASF Outbreak Clusters, Velocity of Spread and Geographic Patterns

**Figure 3:**
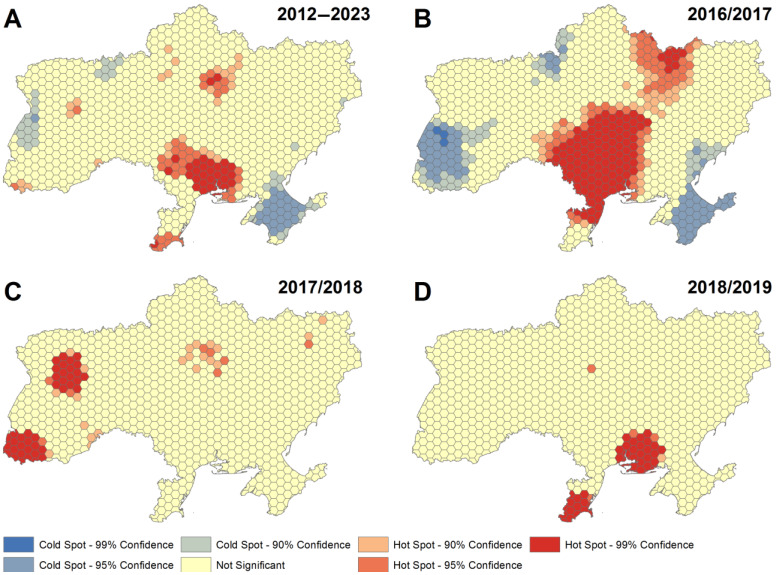
Series of hotspot maps, with colors trending towards red indicating high and trending towards blue low ASF outbreak activity. Panel (**A**) based on all reported cases from 2012 through May 2023; panels (**B**–**D**) by season (April–March) for the years with the highest numbers of ASF outbreaks 2016/2017 (**B**), 2017/2018 (**C**) and 2018/2019 (**D**).

All oblasts reporting to the State Service of Ukraine for Food Safety and Consumer Protection (Crimea and parts of Donetsk and Luhansk were not reporting since late 2014) were affected by ASF, but outbreak events were not equally distributed spatially. Areas with a relatively high incidence of outbreaks fell along a north–south axis in central Ukraine during 2016/2017, in the west during 2017/2018 and in the south during 2018/2019 (Figure 3). Data in the years preceding 2016 and past 2019 were insufficient to generate meaningful heat maps.

**Figure 4:**
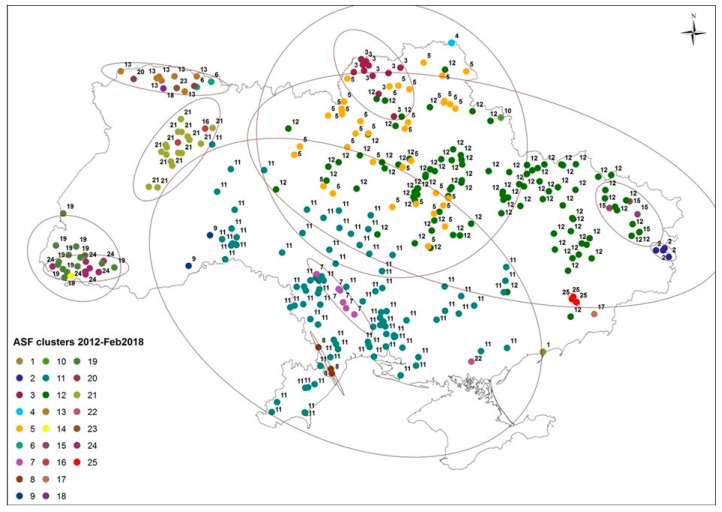
Epidemiological clustering of ASF outbreaks in Ukraine, 2012–2018 (numbered chronologically) according to the “biology and behavioral model”. Ellipses refer to one standard deviation around the mean geographic center of outbreaks.

We conducted a temporal and spatial analysis of the outbreak data using a biology and behavior model with time and distance between outbreaks as inputs. This model revealed at least 25 clusters of ASF outbreaks (2012–2018), with some of the clusters starting with outbreaks in wild boar and others with outbreaks in domestic pigs. Starting in November 2016, there was an increasing number of ASF cases that could not be clearly allocated to any particular cluster due to the temporal (<60 days) and spatial vicinity (<200 km) to outbreaks from two or more earlier clusters. As a consequence, the model was unable to distinguish clusters beyond February 2018 (Figure 4, Figure S3). The estimated velocity of ASF dissemination varied from cluster to cluster, with an average of 5.2 km/day. The velocity of dissemination of clusters starting with a wild boar report was an average of 1.4 km/day slower than the 6.7 km/day determined for clusters starting with a report from domestic pigs. The first cluster with a dissemination velocity exceeding 10 km/day started in 2016 (Table S1).

**Figure 5:**
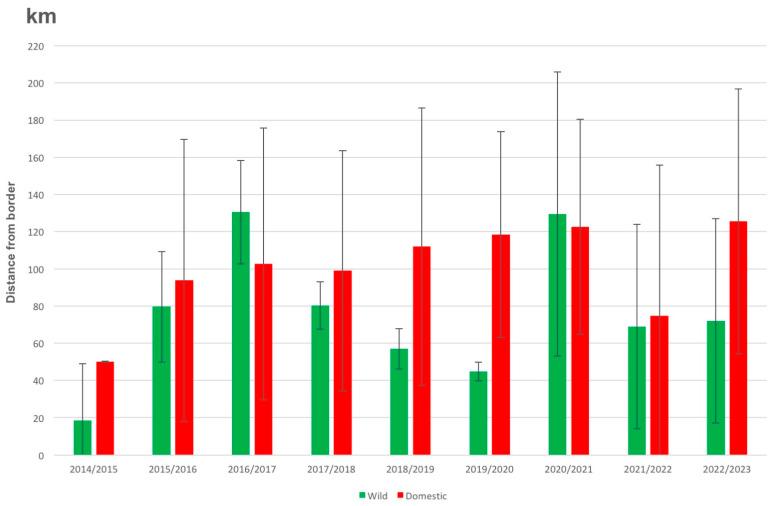
Graph depicting the average distance and standard deviation of outbreaks from official borders or the north of Crimea oblast by season (April–March) for domestic pigs (red) and wild boars (green).

We then evaluated distance of outbreaks from international borders and transportation routes. The average distance of wild and domestic outbreaks from a border increased during the first years of ASF in Ukraine (2014–2017) and has been relatively stable (domestic) or decreasing (wild) from 2017 to 2020 (Figure 5). Despite the trend, distance to an international border was not significant over six annual hunting seasons between wild boar and domestic pig outbreaks (2014/15–2019/20 seasons; F = 2.19, *p* = 0.11, n.s., one-way ANOVA; *p* = 0.17, n.s., Tukey-HSD pairwise statistic). However, distance to a border for wild boar cases each season was marginally closer in comparison to the average distance to borders of all outbreaks from 2014–2020 (F = 3.39, *p* = 0.095, one-way ANOVA), a trend that was significant when wild boar cases were compared to the average distance of all domestic swine outbreaks across all years (F = 5.20, *p* = 0.046, one-way ANOVA). The driving distance from outbreak sites to the next major transit route varied greatly, with an average of 8 km for domestic pigs and an average of 12 km for wild boar outbreaks (n.s.). The next larger city was between <1 and 197 road km (average 58 km) away from domestic pig outbreak sites and between <1 and 214 road km (average 67 km) away from wild boar outbreak sites (n.s.; Figures S4 and S5).

**Figure 6:**
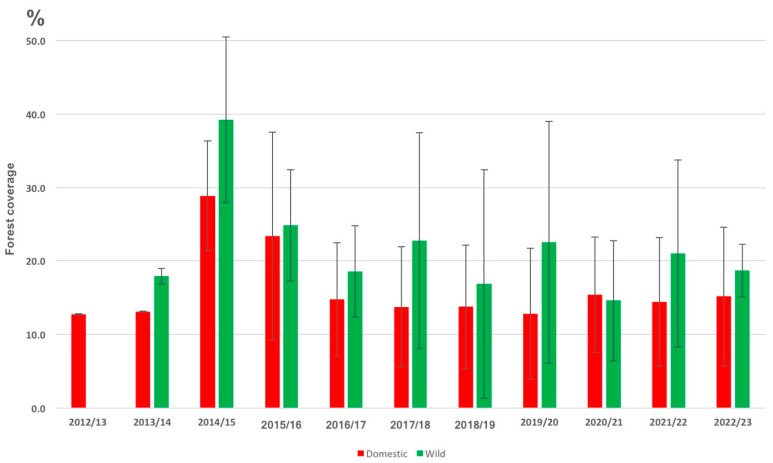
Graph showing the average forest density (circles with a radius of 25 km) around outbreak sites by season (April–March) for domestic pigs (red) and wild boars (green).

Correlation of ASF outbreaks with forest coverage showed that outbreaks in wild boar (2013–2023) correlated with higher percent forest coverage within 25 km of outbreaks than the average for all of Ukraine, a trend that was marginally significant (F = 4.34, *p* = 0.059, one-way ANOVA) (Figure 6). This trend was marked in the 2014/15 hunting season (25–39% forest coverage within 25 km of outbreaks), possibly reflecting introduction and spread of ASFV in wild boar migrations from forested international borders towards the interior of Ukraine (Figure 4). Occurrence of ASF outbreaks in domestic pigs in forested regions also peaked in 2014/15, although the incidence is not significant. The average forest coverage around outbreaks in the following years (2016–2023) was below the average across Ukraine (16.8%) for domestic pigs (13–15%) and above for wild boars (17–23%), with the exception of 2020/21, a period in which only two wild boar cases were recorded (Figure 6).

**Figure 7:**
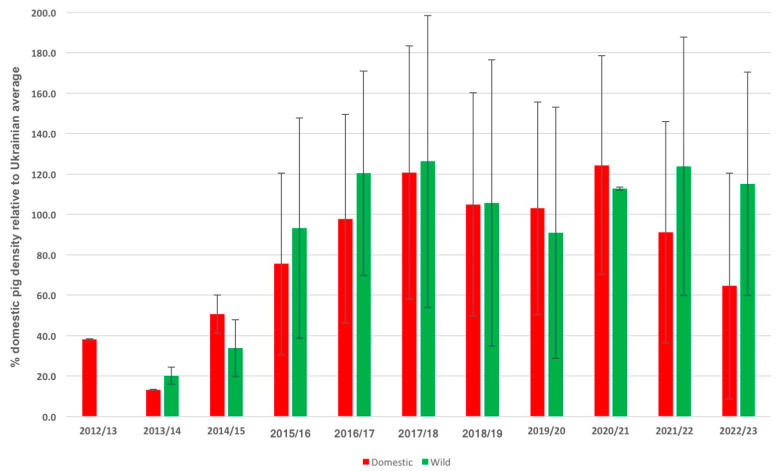
Graph showing the correlation between outbreaks in domestic pigs (red) and wild boars (green) with the modelled population density of domestic pigs in Ukraine in the surrounding area (circles with 25 km radius). Data by season (April–March) and displayed in % of average pig density in Ukraine with standard deviations.

The correlation between the outbreaks and the density of domestic pig population in the area around the outbreak has changed over time. While outbreaks in the early years (2012–2015) that occurred in areas with pig populations were below the average domestic swine population density in Ukraine (0–51%), ASF outbreaks reported from 2016 tended to occur where domestic pig density is closer to the country average (65–126%) (Figure 7). However, the differences between wild boar and domestic pigs and the trend of ASF outbreaks increasingly occurring in regions of higher pig population density did not reach statistical significance.

Of the 566 outbreaks, 125 were found in wild boars, 309 in backyard farms and 95 in commercial farms. Thus, 0.015% of backyard farms and 4.647% of commercial farms in Ukraine have so far reported ASF cases, while ASF detections in wild boar suggest that 2.083% of sounders were affected.

## 4. Discussion

The analysis of the ASF outbreak data in Ukraine revealed several interesting patterns, such as different peak months for reporting of ASF in domestic pigs (August) and wild boars (December). Based on the time and space between reported outbreaks, 25 distinct clusters were identified between 2012 and 2018, but, while the vicinity to borders, forest density and a low density of domestic pigs were predictors up until 2016, this was no longer the case in the more recent years.

While the first reported outbreak of ASF in Ukraine since 1977 dates back to the summer of 2012, it was not until four years later that outbreak reports were indicating a steep increase in cases in Ukraine (Figure 1). Based on the locations of outbreaks reported in the years between 2012 and 2016, as well as on their geographical and temporal distance from each other, we conclude that ASF was probably introduced on multiple occasions before it was able to establish sustained local spread within Ukraine (Figure 4). The data from the ASF biology and behavior model, as presented in Figure 4 (ASF clusters 2–6), suggest that the introduction happened in the periphery along the eastern and northern borders of Ukraine as outbreak locations were generally close to the border before 2016 regardless of whether the outbreaks were detected in wild boar or domestic pigs (Figure 5).

Even though more than 70% of the reported outbreaks before 2015 were in wild boar, it is impossible to determine with certainty if it was the wild boars that were responsible for the introduction of ASF into the country. Inadvertent import and dissemination of the virus with pork products or live animals is a known risk factor for ASF spread [31]. It is not unlikely to have played a role in Ukraine, and provides a likely explanation for the isolated 2012 outbreak in Zaporozhye.

Previous studies have suggested that wild boars are important drivers of ASF spread in the Baltic states and Poland [41,42,43,44,45,46]. However, our analysis suggests a more differentiated picture for the dynamics in Ukraine. The high proportion of wild boar outbreaks during the beginning of the epidemic, in combination with a relatively high percentage of forest cover (>20%), an indicator of potential wild boar habitat, around the outbreaks, even for domestic pigs up until mid 2016, suggest that wild boars have played a role during that phase. However, the speed with which some of the outbreak clusters have unfolded and spread over the country (>10 km/day) makes wild-boar-driven spread being the sole explanation highly unlikely (Table S1) as wild boars tend to be territorial and normally do not migrate or cover large distances during foraging.

The different trends observed for indicators such as forest density (higher for wild boar) and distance to borders (increase then decrease for wild boar, increase and stabilize for domestic pigs) for wild boars versus domestic pigs in general, and for outbreak clusters in particular (different speeds of spread), suggest that ASF spread in wild boars and domestic pigs may have been uncoupled or that human practices have become the main driver since 2016 (Figure 4, Figure 5 and Figure 6). In fact, geographically, we found that, each year, wild boar outbreaks were significantly closer to international borders than the average distance of domestic pig outbreaks to borders across all years (Figure 5). In the 2014/15 hunting season, these detections tended to be in forested regions, reflecting wild boar habitat and hunting practices (Figure 6). The velocity of spread of ASF in wild boars was estimated to be slower than the spread of ASF in domestic pigs (Table S1). This may reflect the natural migratory behavior of wild boar in forested habitats as opposed to human-facilitated spread of ASF among domestic pig farms. A primarily wild-boar-driven ASF spread is typically estimated to move at a speed of 1.5 to 5 km/month based on the data from various countries [27,36,43,47]. Note, however, that the actual incidence of ASF in wild boars in Ukraine is unknown as there is no systematic monitoring of live wild boar populations for ASF or other pathogens. Nevertheless, the speed at which ASF seems to have spread across Ukraine is irreconcilable with a primarily wild-boar-driven transmission. It would have taken ASF 5–20 years to reach the central and southern parts of the country from any of the borders with Russia and Belarus where ASF was detected in 2014, but the first cases were reported as far south as Odessa (oblast) after less than 2 years.

Similar developments where wild boar movement has driven ASF spread initially but then humans have become the major ASF vector have been postulated as the most likely scenarios responsible for ASF spread in the Russian Federation and in Romania [48,49,50]. Our conclusion is also supported by the fact that Ukraine, with its large agricultural planes, offers overall limited habitats for wild boars [51]. With an estimate of around 30,000 animals, Ukraine has a much smaller wild boar population than neighboring Poland, where almost 250,000 wild boars were hunted in 2021 in a country half the size of Ukraine [52]. The large boar population in Poland has been able to sustain a continuous circulation and spread of ASF, with wild boar outbreaks dominating by far; in contrast, only about one quarter of the reported outbreaks in Ukraine were in wild boars [15,52,53].

Neither the wild boar habitats nor the wild boar ASF cases are evenly distributed across Ukraine, and the majority can be found along the northern and western borders. The northern part of Ukraine was indeed where most of the early outbreaks occurred, which would be consistent with, at that stage, mainly wild-boar-driven ASF spread. Because of these difference in habitats as well as to account for potential differences related to climate, we differentiated between the three southern oblasts Kherson, Odesa and Mykolaiv adjacent to the Black Sea and the other primarily more northern regions (Figure 2). A colder climate with low temperatures in the winter could increase ASFV survival in the environment, such as in carcasses, and the data from the northern wild boars indeed show more cases in the cold months (November–February) [30,31,42]. This pattern of seasonality has also been observed in Poland and in Lithuania [44,54].

However, colder temperatures may only be one factor in the equation since the animals’ mating season and the local hunting season fall into the same winter months and may contribute to it equally or more. A pattern of outbreaks consistent with seasonality can also be observed among domestic pigs, where outbreak numbers peak in and around August all over Ukraine. A second peak in the winter (January and February) is interestingly only present in the more northern oblasts, which could indicate a connection between these winter outbreaks in domestic pigs and the high circulation of the virus in winter in wild boars (Figure 2).

The risk assessment based on the holding form (commercial or backyard farm) for domestic pigs and for wild boars in general is certainly only a very rough indicator of risk, but it did reveal some interesting insights. While the assumption that only about 2% of wild boar sounders were affected seems rather low, it might be attributed to two factors, with the first being a smaller potential for transmission due to low population density and the second being an unknown number of undetected or unreported cases in wild boar.

The ratio of inferred risk between backyard farms and commercial farms appears more puzzling, especially in light of knowledge gaps regarding ASF amongst backyard farmers [55]. Considering that commercial farms are likely to adhere to a higher level of biosecurity to prevent contact with wild boars and to keep any potentially contaminated pork products out, it seems counterintuitive that their risk for having an ASF outbreak is in the order of two magnitudes above that of backyard farms. An alternative explanation may be bias in reporting. It is conceivable that a potential lack of awareness, lack of motivation, uncertainty in financial reimbursement or concern for financial security might facilitate underreporting of ASF in backyard farms in Ukraine. Costard previously predicted that exactly such factors may be important drivers for a backyard-farm-driven spread of ASFV [56].

ASF outbreak numbers in Ukraine have been decreasing since 2018, which has led to a flattening of the curve (Figure 1). A similar trend has been observed in other European countries. The Czech Republic had regained the status of being ASF-free but lost the status again in December 2022. Similarly, Estonia had not reported outbreaks in pig farms between October 2017 and July 2021. Presently, most reports of new cases are connected to seropositive wild boars [10,57]. Poland, which is currently the country in Europe most affected by ASF in wild boars, also noticed a decrease in the number of ASF outbreaks in pig farms, which is similar to the situation observed in other Baltic states [54,58].

Whether this trend continues in Ukraine remains to be determined, and it has been suggested that ASF may have become endemic in Ukraine [59]. In 2021, Ukraine reported only thirteen outbreaks in domestic pigs and three cases of ASF in wild boar [ADNS]. It might be assumed that measures taken by governmental and private stakeholders, especially information and sensitization campaigns for backyard farmers and increased biosafety around commercial farms, have contributed to the downward trend in outbreaks. In 2022, seven domestic pig and two wild boar cases have been reported, and two each in 2023 between January and May. However, surveillance for ASF and other reportable veterinary diseases has been negatively impacted by the war in Ukraine (beginning with the invasion in February 2022), which has likely hampered ASF surveillance and control efforts. Moreover, neighboring countries continue to detect ASF, so (re-)introduction remains a major concern, even if local spread can be reduced further or fully stopped (Richter 2023). Vigilance and continued surveillance will thus remain crucial while the quest for an ASF vaccine continues [25].

## 5. Limitations

This work is based on the official data collected in Ukraine by the FSCP and provides evidence for a more differentiated view on ASF introduction and the mode of spread in Ukraine. The study has several limitations, however, due to the nature of the dataset. FSCP was unable to collect data on Ukrainian territory that was not under the control of the Ukrainian government, which includes Crimea and parts of Luhansk and Donetsk since 2014, as well as additional parts of Ukraine that, after February 2022, were temporarily or, as of May 2023, still are under Russian occupation. The dataset covers only reported outbreaks of ASF, which means that the infection chains (clusters) we identified are likely incomplete, especially when it comes to wild boar cases, but likely also for outbreaks in backyard farms. Our biology and behavior model is an attempt to account for that and to help in the understanding of the dynamics that lead to ASF establishing itself in Ukraine. The model fails to provide useful infection chains beyond February 2018 though, due to a high density and frequency of outbreaks. Sequencing of confirmed ASF samples would be the method of choice for determining how individual outbreaks relate to each other, and the work presented here can inform and guide such efforts. As the study was not designed to relate the ASF dynamic with the countermeasures employed by the government, we cannot judge the effectiveness and direct impact of such measures; the steady decrease in outbreaks since 2019, however, suggests at least partial success.

## 6. Conclusions

Based on the distances and intervals between reported ASF cases prior to 2016, we conclude that ASFV was likely introduced to the territory of Ukraine from neighboring countries on multiple occasions. Introduction, spread and maintenance of the virus may have initially been driven by the wild boar population to a large extent, but the distribution of outbreak reports in more recent years suggests a shift to a mode of spread that is driven by human activity. Mitigation strategies have shown success in reducing the ASF burden in Ukraine.

## Data Availability

Source data available at https://www.asf.vet.ua/ (accessed on 8 May 2023).

## Supplementary Materials

The following supporting information can be downloaded at: https://www.mdpi.com/article/10.3390/vaccines11071145/s1, Figure S1: Map of Ukraine depicting climate-based separation of oblasts; Figure S2: Map of all ASF outbreaks in Ukraine; Figure S3: Space-time permutation model of ASF outbreaks; Figure S4: Outbreak distance from major roads; Figure S5: Outbreak distance from major cities; Table S1: Dissemination of ASF in Ukraine.
