# Supplementary material for: Epidemiological Characterization of African Swine Fever Dynamics in Ukraine, 2012–2023
Source: Vaccines (Basel). 2023 Jun 25;11(7):1145. doi: 10.3390/vaccines11071145 (PMC10384127; doi:10.3390/vaccines11071145)

Supplement 1

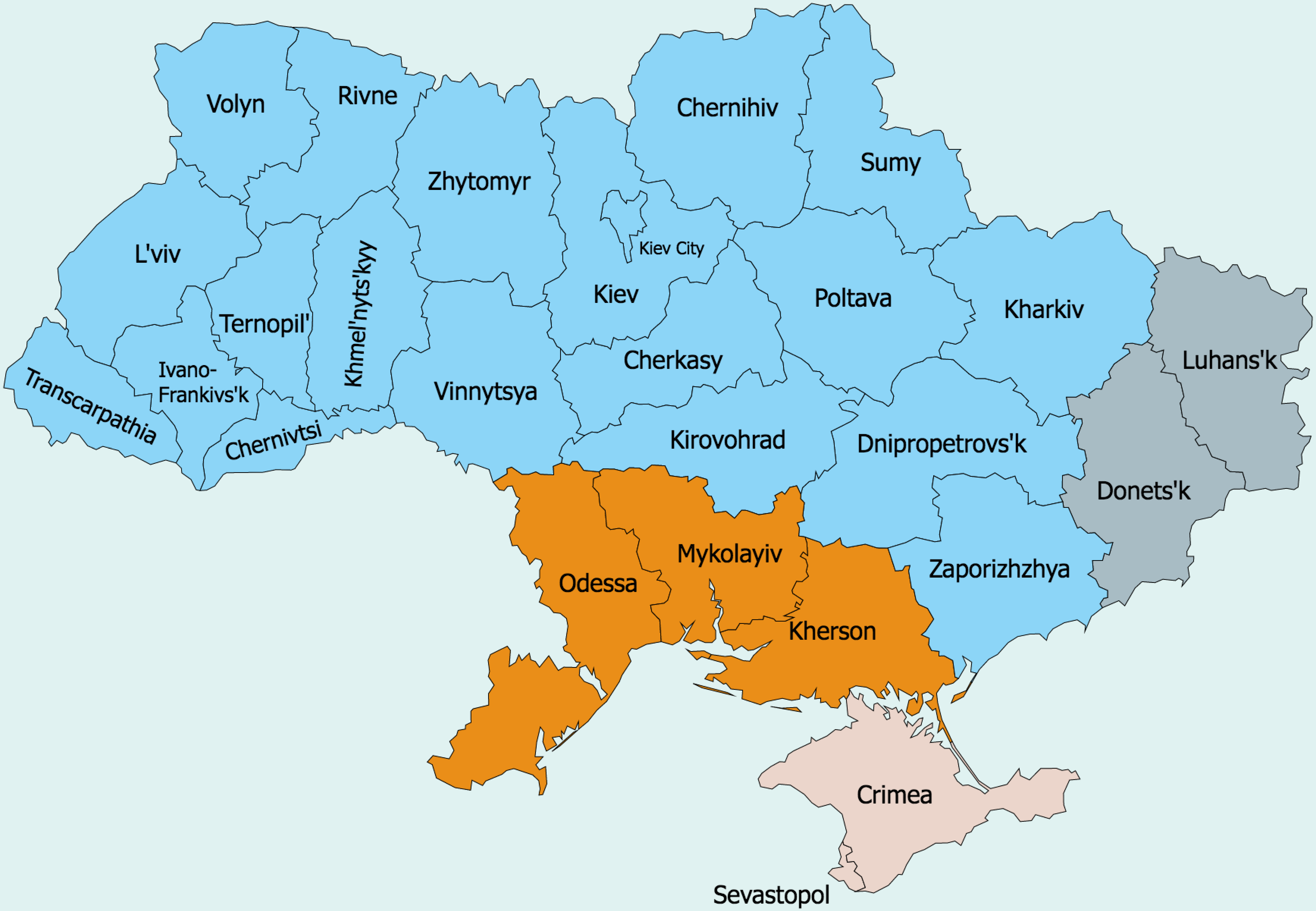

# Supplement 2

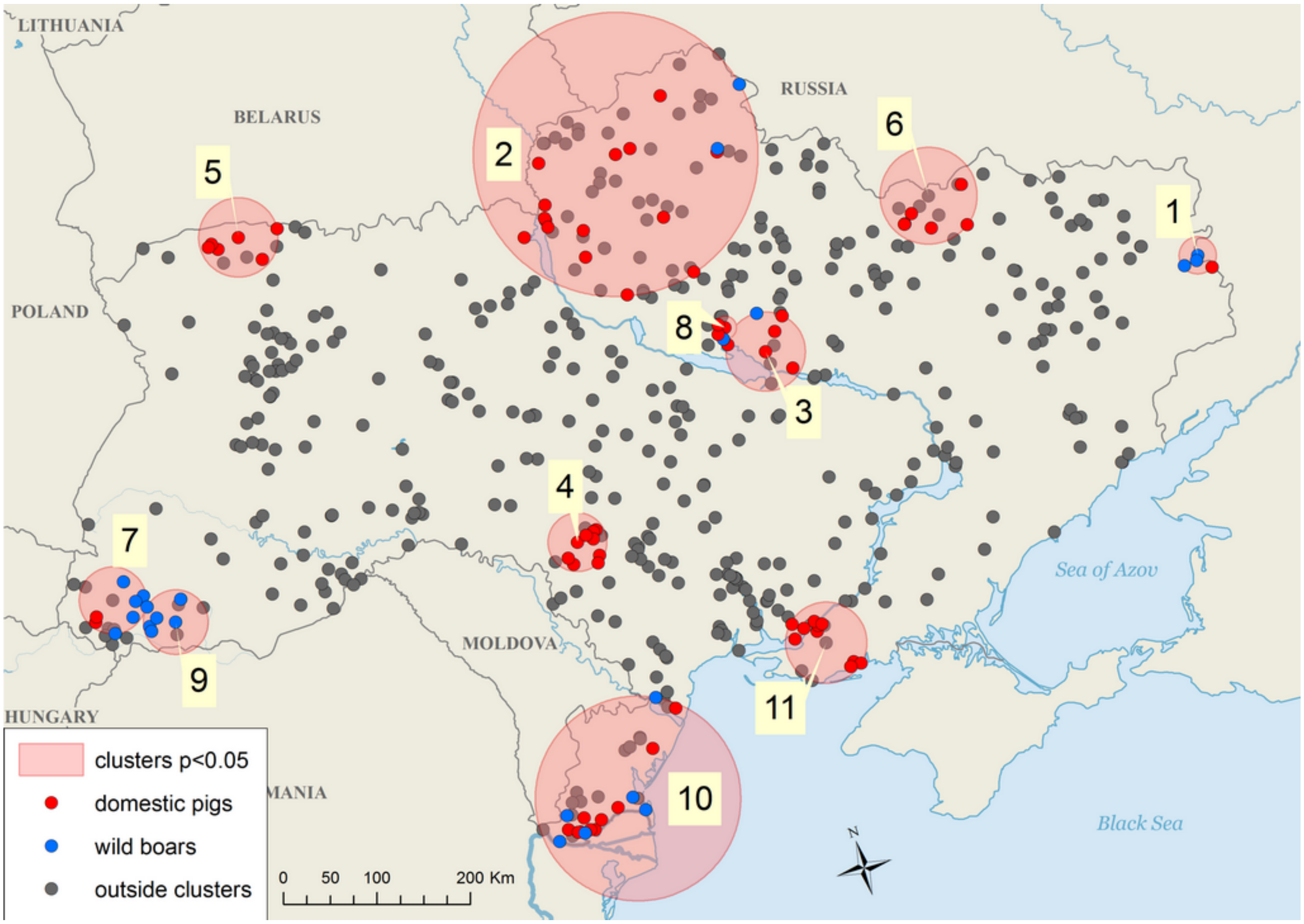


Space-time permutation model of ASF outbreaks in Ukraine 2012-Jun2020. Red circles represent space-time clusters. The clusters are numbered in time order when they occurred. Colored dots represent locations with observed outbreaks during the time dimension of the cluster. Black dots represent outbreaks which less likely have space-time interaction.

| Cluster | Radius, km | Start date | End date | P-value | Observed | Expected |
|---|---|---|---|---|---|---|
| 1 | 20.1 | 1/6/2014 | 2/16/2014 | 4.66E-04 | 4 | 0.03 |
| 2 | 153.8 | 6/24/2015 | 8/20/2015 | 3.08E-07 | 17 | 2.04 |
| 3 | 42.8 | 12/17/2015 | 2/12/2016 | 3.41E-05 | 6 | 0.12 |
| 4 | 31.7 | 7/26/2016 | 8/15/2016 | 8.01E-06 | 9 | 0.44 |
| 5 | 42.9 | 8/18/2016 | 8/23/2016 | 9.24E-05 | 6 | 0.14 |
| 6 | 52.3 | 8/26/2016 | 10/7/2016 | 4.00E-02 | 6 | 0.32 |
| 7 | 36.1 | 7/5/2017 | 8/21/2017 | 2.20E-02 | 7 | 0.46 |
| 8 | 12.4 | 7/7/2017 | 7/20/2017 | 1.20E-02 | 4 | 0.06 |
| 9 | 34.9 | 2/1/2018 | 2/1/2018 | 2.22E-04 | 6 | 0.16 |
| 10 | 110.2 | 6/21/2018 | 7/27/2018 | 5.91E-08 | 16 | 1.61 |
| 11 | 43.7 | 7/13/2018 | 9/8/2018 | 7.85E-03 | 10 | 1.09 |

Description from the SatScan user guide from https://www.satscan.org/techdoc.html:

**Space-Time Permutation Model**

The space-time permutation model requires only case data, with information about the spatial location and time for each case, with no information needed about controls or a background population at risk. The number of observed cases in a cluster is compared to what would have been expected if the spatial and temporal locations of all cases were independent of each other so that there is no space-time interaction. That is, there is a cluster in a geographical area if, during a specific time period, that area has a higher proportion of its cases in that time period compared to the remaining geographical areas. This means that if, during a specific week, all geographical areas have twice the number of cases than normal, none of these areas constitute a cluster. On the other hand, if during that week, one geographical area has twice the number of cases compared to normal while other areas have a normal amount of cases, then there will be a cluster in that first area. The space-time permutation model automatically adjusts for both purely spatial and purely temporal clusters. Hence there are no purely temporal or purely spatial versions of this model.
*Example:* In the space-time permutation model, cases may be daily occurrences of ambulance dispatches to stroke patients.
It is important to realize that space-time permutation clusters may be due either to an increased risk of disease, or to different geographical population distribution at different times, where for example the population in some areas grows faster than in others. This is typically not a problem if the total study period is less than a year. However, the user is advised to be very careful when using this method for data spanning several years. If the background population increases or decreases faster in some areas than in others, there is risk for population shift bias, which may produce biased p-values when the study period is longer than a few years. For example, if a new large neighborhood is developed, there will be an increase in cases there simply because the population increases, and using only case data, the space-time permutation model cannot distinguish an increase due to a local population increase versus an increase in the disease risk. As with all space-time interaction methods, this is mainly a concern when the study period is longer than a few years180,182. If the population increase (or decrease) is the same across the study region, that is okay, and will not lead to biased results.

**Supplement 3:** Dissemination of ASF in Ukraine

| **Cluster*** | **First report** | **Max distance** *in km* | **Speed** *in km/day* | **First case in** |
|---|---|---|---|---|
| 2 | 2014-01-06 | 20 | 0.8 | wild boar |
| 3 | 2014-08-26 | 96 | 2.4 | domestic pig |
| 5 | 2015-05-05 | 276.4 | 3.6 | wild boar |
| 6 | 2015-07-02 | 29.6 | 0.5 | wild boar |
| 7 | 2015-10-24 | 116 | 4.0 | domestic pig |
| 8 | 2016-03-18 | 63.1 | 3.0 | domestic pig |
| 9 | 2016-05-10 | 77.3 | 2.8 | domestic pig |
| 11 | 2016-06-24 | 399.1 | 2.7 | domestic pig |
| 12 | 2016-06-28 | 417.4 | 3.8 | domestic pig |
| 13 | 2016-08-18 | 129.7 | 16.2 | domestic pig |
| 16 | 2017-02-06 | 60.7 | 4.7 | domestic pig |
| 18 | 2017-04-19 | 124.9 | 2.8 | domestic pig |
| 19 | 2017-05-29 | 169.9 | 1.6 | wild boar |
| 20 | 2017-08-15 | 118.1 | 23.6 | domestic pig |
| 26 | 2017-02-18 | 25.4 | 0.7 | wild boar |
| 27 | 2018-05-05 | 52.1 | 2.9 | domestic pig |
| 28 | 2018-06-10 | 91.3 | 11.4 | domestic pig |
| | | | | |
| **Overall Ø** | - | **133.4** | **5.2** | - |
| | | | | |
| **Wild boar Ø** | - | **104.3** | **1.4** | - |
| | | | | |
| **Domestic pig Ø** | - | **145.5** | **6.7** | - |

* Compare Figure 4

# Supplement 4

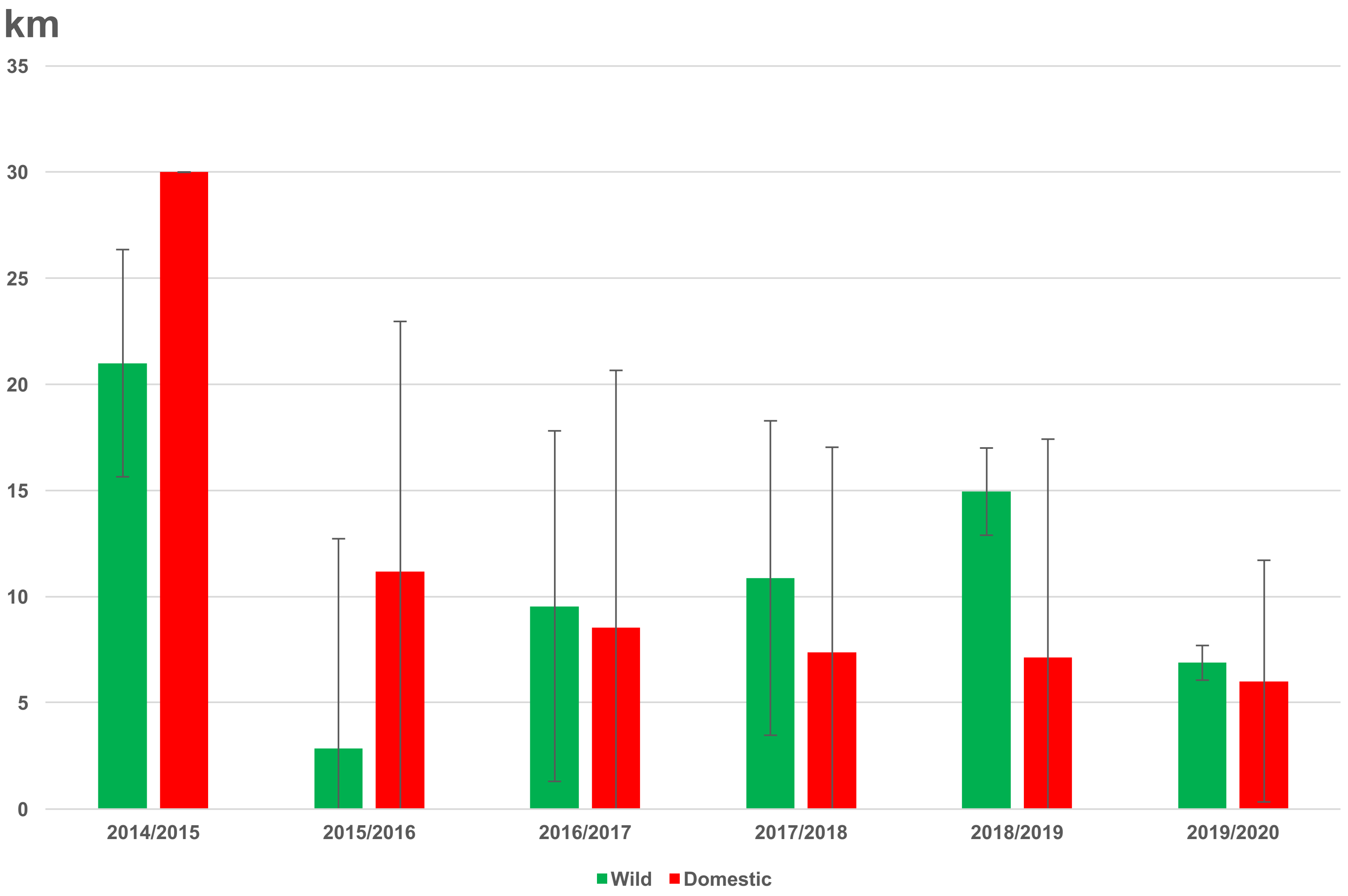

# Supplement 5

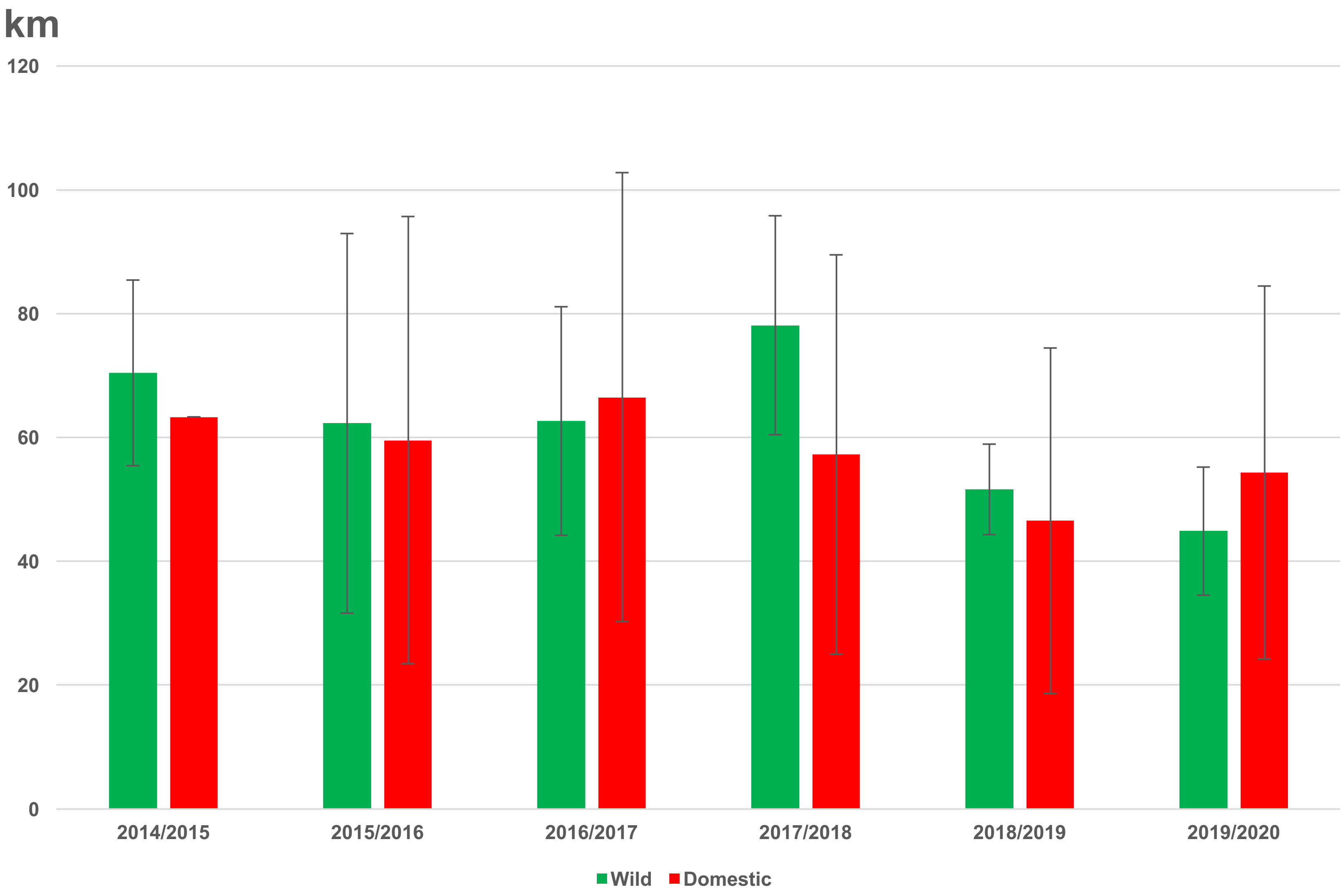

Supplement: Supplementary file 1 [file vaccines-11-01145-s001.zip › vaccines-2350102-supplementary.pdf]
